# The distinct roles of reinforcement learning between pre-procedure and intra-procedure planning for prostate biopsy

**DOI:** 10.1007/s11548-024-03084-4

**Published:** 2024-03-07

**Authors:** Iani J. M. B. Gayo, Shaheer U. Saeed, Ester Bonmati, Dean C. Barratt, Matthew J. Clarkson, Yipeng Hu

**Affiliations:** 1 Department of Medical Physics and Biomedical Engineering, University College London, London, UK; 2 Wellcome/EPSRC Centre for Interventional and Surgical Sciences, University College London, London, UK; 3 Department of Computer Science and Engineering, University of Westminster, London, UK

**Keywords:** Reinforcement learning, Biopsy, Planning, Prostate cancer

## Abstract

**Purpose:**

Magnetic resonance (MR) imaging targeted prostate cancer (PCa) biopsy enables precise sampling of MR-detected lesions, establishing its importance in recommended clinical practice. Planning for the ultrasound-guided procedure involves pre-selecting needle sampling positions. However, performing this procedure is subject to a number of factors, including MR-to-ultrasound registration, intra-procedure patient movement and soft tissue motions. When a fixed *pre-procedure planning* is carried out without intra-procedure adaptation, these factors will lead to sampling errors which could cause false positives and false negatives. Reinforcement learning (RL) has been proposed for procedure plannings on similar applications such as this one, because intelligent agents can be trained for both pre-procedure and *intra-procedure planning*. However, it is not clear if RL is beneficial when it comes to addressing these intra-procedure errors.

**Methods:**

In this work, we develop and compare imitation learning (IL), supervised by demonstrations of predefined sampling strategy, and RL approaches, under varying degrees of intra-procedure motion and registration error, to represent sources of targeting errors likely to occur in an intra-operative procedure.

**Results:**

Based on results using imaging data from 567 PCa patients, we demonstrate the efficacy and value in adopting RL algorithms to provide intelligent intra-procedure action suggestions, compared to IL-based planning supervised by commonly adopted policies.

**Conclusions:**

The improvement in biopsy sampling performance for intra-procedure planning has not been observed in experiments with only pre-procedure planning. These findings suggest a strong role for RL in future prospective studies which adopt intra-procedure planning. Our open source code implementation is available here.

## Introduction

### MR-targeted TRUS-guided prostate biopsy procedures

Acquiring magnetic resonance (MR) imaging scans is recommended before previously commonly adopted “blind” biopsy of prostate cancer [1]. In recent years, pre-procedural MR reporting has increasingly been followed by an MR-targeted approach for biopsy of suspicious lesions found on MR, typically guided by intra-procedural transrectal ultrasound (TRUS) imaging. A brachytherapy template can also be adopted to assist with sampling to stabilise needle insertion. To prepare for such a procedure, clinicians utilise pre-procedural MR scans with annotated positions of multiple suspected lesions to obtain an approximate plan of where to sample needles.

The most intuitive sampling strategies devised before the biopsy include sampling template grid positions closest to the lesion centre. However, applying this strategy during the procedure is subject to several potential targeting errors that result in false negatives from missed tumour samples [2]. First, the mapping of the pre-operative positions to the live US-images of the prostate requires cognitive or computational registration, both of which are subject to registration error [3]; second, the patient is subject to movement during the procedure; third, organ motion occurs due to factors such as respiratory motion, bladder filling, the US probe movement, needle insertion and non-trivial needle bending [4]. These errors are not independent of each other and may be difficult to de-couple, which means that targeting imprecision may be a result of a mixture of these. Their collective effect can be understood and modelled by a non-rigid spatial mismatch between MR-informed targets and actual biopsy sampled locations. Given the presence of these spatial mismatches, there is a need to learn robust and adaptable sampling strategies based on observed intra-procedure changes.

### Related work

Previous work demonstrated that strategies which sample biopsy needles only at the centre of the lesion may lead to under-grading of tumours, especially in the case of heterogeneous PCa [5]. The study suggests that targeting the peripheral of the lesions could lead to more representative samples of the suspected lesions. However, to the best of our knowledge, there are limited studies that quantify the effectiveness of this strategy, or that search for alternative or optimum sampling strategies. In [6], reinforcement learning (RL) is used to learn patient-specific targeting strategies. An interesting finding is that RL learned strategies that adapted to the size of the lesions, by spreading the needles more for smaller lesions, achieving a similar detection rate as larger lesions. However, key limitations in training RL models are highlighted in this work: long training times, especially when training individual patient models, and sample-inefficiency as large amounts of experiences are required before learning an optimal strategy. These findings suggest a need to speed up the training process of RL, before training models that can generalise to multiple unseen cases.

Outside of prostate biopsy, RL has also been successfully applied in similar needle planning applications. In [7], RL is used to learn optimal electrode trajectories for thermal ablation of liver tumours. An environment is constructed to simulate the procedure, using fixed 3D masks derived from CT scans. To guide the agents’ learning, rewards are devised based on clinical constraints relevant to the ablation process, such as ensuring the electrode trajectories do not collide with organs of interest. They report results based on mean accuracy and failure cases, based on whether the trajectory is able to reach the target tumour. However, it is unknown whether their method is able to adapt to changes which may occur intra-operatively, such as tissue deformation or needle bending, which were not included in their simulations. In [8], RL is used for planning flexible needle insertion paths for surgical robotics. The agent learns actions that rotate and move the direction of insertion of the needles, receiving negative rewards for colliding with organs at risks. In their simulations, uncertainty from flexible needle-tissue interactions are modelled through a stochastic environment which incorporates a 10$\%$ probability for movement failure. Their results suggest that the agent is able to learn optimal trajectories regardless of these uncertainties, achieving a high number of success cases. Thus simulation of these uncertainties could prove beneficial, allowing agents to learn adaptive and robust strategies.

### RL for pre-procedure and intra-procedure planning

In this work, we propose to train RL agents that suggest a sequence of sampling locations without utilising previous intra-procedure sampling steps. We define this type of plan as “pre-procedure planning”. In this case, the RL agent acts as a trial-and-error optimisation approach to find optimal sampling location distribution, and potentially the optimum order of them, before the procedure.

Another potential advantage of using RL includes modelling the dynamic decision making process — data from previous steps can be used to suggest the next sampling actions. The sequential data modelling nature of RL algorithms enables real-time and potentially better action suggestion on-the-fly, which hereinafter is referred to as “intra-procedure planning”. The key difference is the availability of intra-procedure data, that enables the agents to modify the strategy intra-operatively.

To train the models, training data can ideally be acquired from interaction between agent-suggested actions and observations from the real clinical environment, for off-policy training 1 of intra-procedure planning agents. However, in practice these interactions are acquired through simulating the RL environment. In our work, the patient anatomy and pathology locations are obtained from the pre-procedure MR images and the spatial mismatch is quantified by assumptions on the above-discussed registration error, patient movement and organ motion. Intuitively, the more realistic these simulations are, the better chance the trained agents generalise in the intra-procedure planning. Hence we test the RL-learned strategies in the presence of these errors, to evaluate its potential in being used as an intra-procedure planning tool.

Training RL agents is considered more challenging due to potentially sensitive hyperparameter and algorithm choices; the off-policy interaction is logistically expensive to acquire; and simulating these spatial mismatch factors also requires further research. Therefore we aim to quantify the benefits of intra-procedure planning to justify the use of RL algorithms and interaction data. Furthermore, to address RL’s problem of long-training times, supervised imitation learning (IL) can also be used to train models for both pre-procedure and intra-procedure planning, supervised by “demonstrations” of expert-defined (pre-procedure or intra-procedure) sampling strategies. Combining IL and RL, by initialising RL agents with strategies learned by IL, allows for faster learning with fewer number of interactions required for training, as demonstrated by works in [9] and [10]. However, it is unknown which of these strategies can lead to better sampling performance. Thus we aim to evaluate and compare the performance of agents trained with IL, RL and IL+RL combined.

### Study aim and contributions

In this work, we design a set of experiments to quantify biopsy outcomes using (a) IL-based pre-procedure planning, (b) RL-based intra-procedure planning and (c) training with varying spatial mismatch levels, for quantifying the difference between pre-procedure and intra-procedure planning, with increasing interaction data.

The key contributions of this paper are summarised as follows: (1) we describe a multi-patient training strategy to learn a generalisable sampling strategy that can be applied to unseen patients—for the first time in this application; (2) we demonstrate that initialising RL agents with demonstrated actions of biopsy needle sampling is beneficial for pre-procedure planning in terms of clinically relevant metrics such as hit rate and average cancer core length; (3) we present an interesting observation: training with RL alone is more robust to changes observed intra-operatively such as organ deformation, compared to initialised strategies using demonstrations. Importantly, this suggests the capability of RL in learning novel solutions that are adaptive to intra-procedure changes, making it more suitable for intra-procedure planning.

## Methodology

### Data set

Multiparametric MR images (mpMRI) from 567 patients, with a mixed cohort of biopsy and focal therapy patients, were obtained from multiple clinical trials, including PROMIS [1], SmartTarget [11], and PICTURE [12]. All patients provided written consent, with ethics approved as part of their respective clinical protocols. A detailed description of the entire mpMRI data set, along with applied pre-processing methods such as re-sampling and normalisation, can be found in [13]. In this work, labelled prostate gland and suspected lesion masks are used, which are annotated by radiologists on the T2-weighted sequences. All lesions used in this data set are for lesions with Likert-scores $\ge$ 3. To prevent data leakage, the data set is split at the patient level between training, validation and test sets as 396:58:113. For patients with multiple images obtained at different time points, both images are included within the same split, such that the test set does not include images from the same patients used for training. For each patient, at least two lesion masks are present and treated as individual cases, resulting in 966, 141 and 275 lesions for the training, validation and test sets, respectively. All lesions from the same patient are in the same split. The binary labelled masks of lesions and prostate glands are the only input required for the presented planning strategies in this study.

### Transperineal prostate biopsy procedure

**Fig. 1:**
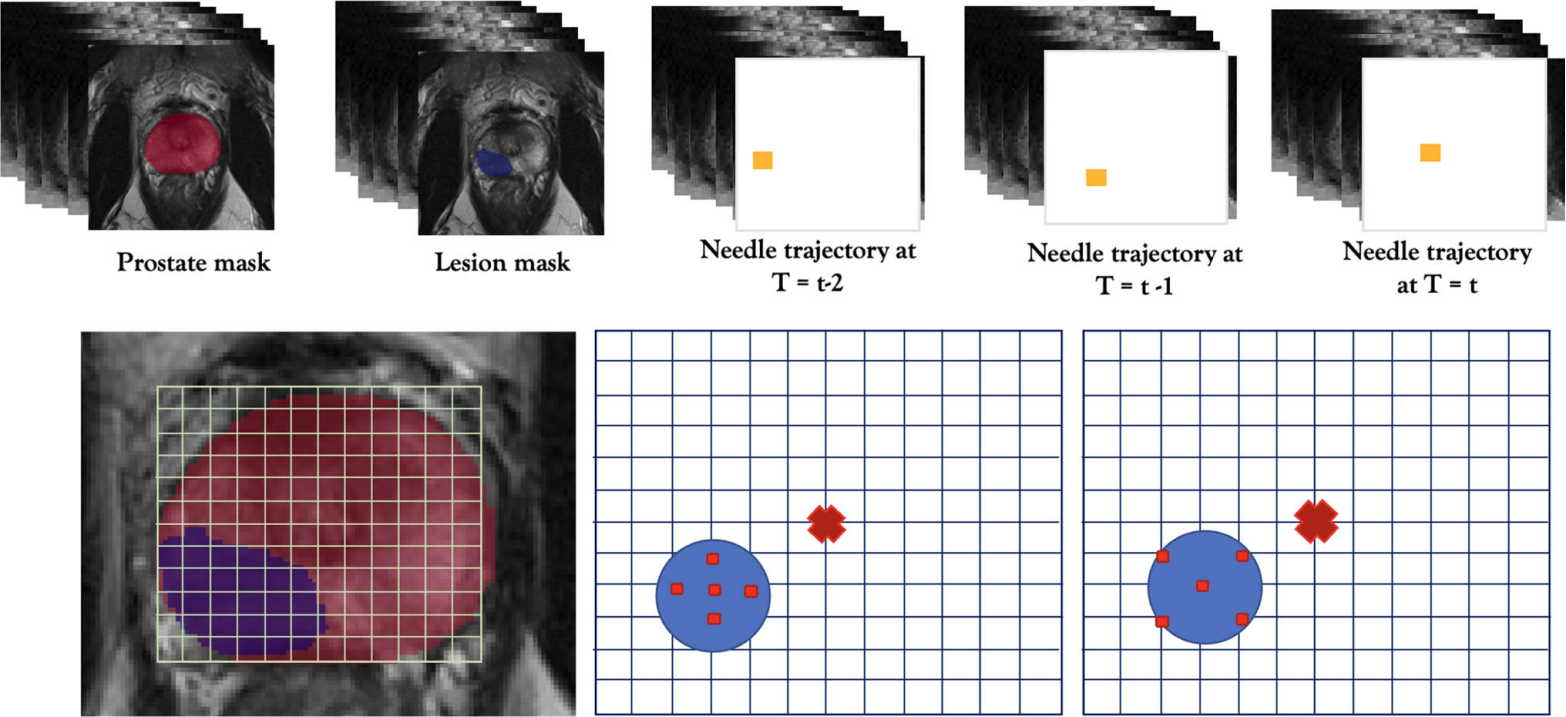
Top row: states $s_t$ provided to agent at time *t*. These consist of the prostate and lesion masks, and needle trajectories for three time steps. Bottom row: left) Template-guided procedure; centre) Centre policy $\pi _\textrm{Centre}(a_t, s_t)$. right) Edge targeted policy $\pi _\textrm{Edge}(a_t, s_t)$. Blue circle represents a target lesion, whilst red squares represent needle core positions. A red cross represents the centre of the template grid

We simulate a targeted transperineal template-guided biopsy procedure using information derived from T2-weighted MR images and their corresponding prostate and lesion masks using the described data set in 2.1. MR images provide the information about the position of the lesion within the prostate gland, and a simulated brachytherapy template grid will be used for needle insertion, for which there are 13x13 discrete locations in a para-transverse plane, which is illustrated in Fig. 1.

Templates are also commonly used in saturation-biopsy, where the entirety of the prostate gland is sampled. In this application we focus on targeted sampling and use the template as a guide for improved stabilised needle insertion. The aim is to learn a sampling strategy that determines the position of five needle positions to target the lesion of interest identified on MR scans.

### Reinforcement learning for optimal core positions

The task of finding optimal biopsy needle positions is formulated as an RL problem, which can be formally defined as a Markov decision process (MDP) tuple:

1 $$\begin{aligned} MDP = <S, A, R> \end{aligned}$$

where *S*, *A* and *R* are the domains of states, actions and rewards, respectively. The state at time-step *t* describes the information provided to the agent, denoted as $s_t \in S$, where $s_t = (P_t, L_t, N_t, N_{t-1}, N_{t-2})$. $P_t$ and $L_t$ describe the prostate and lesion masks respectively. $N_t$ describes a 3D binary mask of the needle trajectory and a history of past trajectories are included, from $N_{t-2}$ to $N_t$, to provide information about previous grid positions, enabling agents to make dynamically informed decisions for next actions. An example of the observed states are illustrated in Fig. 1.

*A* describes the domain of actions that the agent takes within the environment, denoted as $a_t \in A$ where $a_t= (\delta _x, \delta _y, \delta _z)$. The actions $\delta _x$, $\delta _y$ represent continuous movement within the grid between $(-10,+10)$ which are converted into 5 mm intervals, whilst $\delta _z$ represents the depth of needle firing within the patient, for which one out of two depths is chosen: one near the apex and one near the base of the prostate gland. Continuous actions enable the movement of multiple grid positions at a time, allowing for greater exploration whilst reducing the training time required due to the large action spaces.

2 $$\begin{aligned} R = {\left\{ \begin{array}{ll} +10 &{} \textit{if needle fired, intersects with target}\\ -2 &{} \textit{if needle fired, misses target}\\ -1 &{} \textit{if not fired} \\ -5 &{} \textit{if needle is placed outside prostate gland} \end{array}\right. } \end{aligned}$$

The rewards $r(s_t,a_t)=R_t$ can be defined in Eq. 2. Integer rewards are chosen empirically for effective and efficient RL training.

### Policy learning

Using the described MDP, we aim to learn a policy $\pi (\cdot | s_t; \theta )$ that maps a state $s_t$ to action $a_t$ by maximising the expected cumulative sum of rewards $Q^{\pi }(s_t, a_t) = \sum _{k=0}^T\gamma ^k R_{t+K}$, where $\gamma$ is a discount factor applied to balance the contributions of future rewards with intermediate rewards. We denote $Q^{\pi }(s_t, a_t)$ as the Q-value which describes the expected reward received being in a state $s_t$ and taking action $a_t$. Neural networks can be used to parameterise the policy as $\pi (\cdot | s_t; \theta )$ and Q-value network as $Q^{\pi }(s_t, a_t; w)$. An action can be sampled from this policy using $a_t\sim \pi (\cdot | s_t; \theta )$ to provide the sequence of biopsy core positions to target within the grid.

The policy-gradient based algorithm proximal policy optimisation (PPO) is used to learn the optimal parameters $$\theta $$ and *w* for the policy and value-network because of its guaranteed monotonic reward improvement and improved training stability [14]. PPO minimises the loss function in Eq. 3. $$ L_t^{CLIP}$$ is a clipping function that prevents large policy updates, enabling stability in training; $$L_t^{VF}$$ describes the loss between the estimated values from $$Q_\theta ^\pi (s_t,a_t)$$ and actual values obtained from trajectory estimates, whilst H is an entropy term that encourages the agent to visit and explore other states during training. The terms $$c_1$$ and $$c_2$$ are weighting factors describing the contribution of each term in the overall loss function. The optimal policy can be obtained by minimising the combined terms as $$\pi ^* = arg\min _{\theta ,w}(L_t^{CLIP + VF + H})$$. Further details and description of each loss term can be found in [14].

3 $$\begin{aligned}{} & {} L_t^{CLIP + VF + H}(\theta ,w) = {\mathbb {E}}_t [L_t^{CLIP}(\theta )\nonumber \\{} & {} - c_1L_t^{VF}(w) + c_2H[\pi _\theta ](s_t)] \end{aligned}$$

### Learning from demonstrations

Imitation learning (IL) is proposed to address the long training times of RL. We hypothesise that learning from prior demonstrations can help to initialise and guide the exploration of RL agents. We describe a set of demonstrations $D \in (s_0, a_0, s_1, a_1,..., s_n, a_n)$ that consists of paired states *s* and corresponding actions *a* provided by an expert policy. The goal of IL is to imitate these demonstrated actions by learning a policy ${\widetilde{\pi }}_{\theta }(a_t | s_t)$ that closely resembles the demonstrated policy $\pi _{D}(a_t | s_t)$. This involves solving the optimisation problem: ${\widetilde{\pi }}^* = arg\min (L(\pi (a_t, s_t), \pi ^D(a_t, s_t)))$.

Where *L* is a loss function we aim to minimise that compares the two policies. The loss function $L = \frac{1}{n}\sum _{i=1}^{n}(a_{d_i} - a_{pred_i})^2$ describes the mean squared error between predicted actions $a_{pred}$ and demonstrated actions $a_d$ taken at the same state *s*.

We describe two different expert policies that are suggested in clinical practice, which can be visualised in Fig. 1. $\pi _{Centre}(a_t, s_t)$ targets five grid positions closest to each lesion centre. $\pi _{Edge}(a_t, s_t)$ targets the centre of the lesion, followed by four grid positions at the edge of the lesion, similar to a strategy proposed by [5].

### Simulation of intra-procedure mismatches

To investigate the robustness of the agents to registration effects, Gaussian noise is added to the positions of both the prostate and lesion, thereby simulating movement within the observed states, following the previous work [6]. The scale of Gaussian noise added is varied to simulate different levels of target registration error (TRE), which are in line with levels of registration errors reported in previous studies [15].

To devise a controlled organ deformation, a free-form deformation model of the prostate gland is implemented based on [16]. The control points are placed in a 10$\times$10$\times$10 equidistant grid positions over the MR image space, interpolating a smooth transformation using Gaussian splines, for the capability to model both global and local deformations [16]. An efficient PyTorch implementation, based on transposed convolution, is implemented to cope with training large number of on-the-fly sampled deformations and image re-sampling. Varying levels of deformation can be adjusted by two parameters: a rate (the percentage of control points deformed at each time step), and a scale (the range of sampled distance in mm assigned to displaced control points) at each time step. Further details of the adopted deformation model may refer to the provided open-source repository.

## Experiments

### IL and RL implementation and training

Five different models are trained using the same size and network architecture for the policy and value networks, with detailed implementation of the networks found in our open-source repository here. Two IL methods are trained following a supervised-learning approach(Sect. 2.5); 1000 epochs are used for training using an Adam optimiser with learning rate 0.0005. Three RL agents are also trained using the PPO algorithm, two of which are initialised with the weights obtained from IL and one with randomly initialised weights. For training RL models, training lasts for 100,000 episodes, here each episode is an interaction with a new patient data set; the length of each episode is limited to a maximum number of 20 time steps but can terminate early if five fired needles intersect with the lesion. Every 100 episodes, the models are evaluated on validation patients and saved if the average episode reward is higher than previously observed reward values. Similar to IL, an Adam optimiser is used for training but with a learning rate of 0.00005 as a lower rate lead to better training reward convergence. All models are trained using a Ubuntu 18.04.6 operating system, with a Quadro P5000 GPU with 32GB of memory and left for approximately 2–3 days to train until convergence.

**Table 1:** Performance of trained models evaluated on *testing* data consisting of 113 patients and 275 lesion cases altogether

Model	HR (100%)	CCL (mm)	N.CCL	N.Coverage	CCL coeff
$\pi _\textrm{Centre}$	0.418 ± 0.283	4.586 ± 4.606	0.192 ± 0.189	0.959 ± 0.846	0.786
$\pi _\textrm{Edge}$	0.204 ± 0.173	1.916 ± 2.433	0.095 ± 0.104	**2**.**189** ± **1**.**853**	0.780
$\pi _\textrm{Centre}$+RL	**0**.**464** ± **0**.**246**	**4**.**857** ± **4**.**421**	**0**.**231** ± **0**.**173**	0.937 ± 0.765	**0**.**816**
$\pi _\textrm{Edge}$+RL	0.412 ± 0.266	4.503 ± 4.318	0.191 ± 0.175	1.091 ± 0.836	0.761
RL	0.415 ± 0.229	4.298 ± 3.952	0.206 ± 0.170	0.956 ± 0.723	0.786

Best performing results are in bold

### Metrics for biopsy outcome measurements

To measure the performance of these learned strategies, clinically relevant metrics are used which are commonly reported for biopsy outcome. **Hit rate (HR)** is the percentage of needles fired by the agents which hit the lesion. **Cancer core length (CCL)** describes total length of intersection between the needle trajectory and the suspected lesion in mm. We also report another measure ***N.CCL*** which is CCL normalised by the maximum possible CCL obtainable within each volume. ***N.Coverage*** measures the spread of the needles normalised by the area of the lesion, obtained as $\frac{std_x * std_y * \pi }{Area_L}$ where $std_x$ and $std_y$ describes the standard deviation of the *x* and *y* positions of the fired needles, whilst $area_L$ is the 2D area of the lesion projected onto the transverse plane. **Cancer core length correlation coefficient (CCL coeff)** measures the correlation between the lesion size in voxels and measured CCL. This measures how well the sampling strategy can measure the extent of disease burden; a higher coefficient indicates that the obtained CCL is more representative of the true size of each lesion.

### Comparative analysis

To test the statistical significance of the difference in results, paired student’s t-tests were conducted using a significance level of $\alpha =0.05$. We compare the following:

*Comparison of initialised and randomly-initialised agents*—We compare IL models, defined as $\pi _\textrm{Centre}$ and $\pi _\textrm{Edge}$, with RL models, $\pi _\textrm{Centre}+RL$, $\pi _\textrm{Edge}+RL$ and *RL*. This is to measure the benefit of initialising RL with predefined IL-learned strategies, against the benchmark of RL with random initialisation. It also quantifies the potential value in using RL in this application, compared with alternative supervised IL methods. We also qualitatively compare the learned strategies.

*Comparison of RL agents under varying registration errors* For the RL-trained models, we evaluate the performance under different levels of simulated TREs (Sect. 2.6) from 0 to 10 mm. This set of experiments evaluates the sensitivity of RL performance to a specific type of intra-procedure mismatch, likely caused by registration between a MR-based pre-procedure planning and intra-procedure target locations.

*Comparison under varying intra-procedure mismatch* We vary the rates and scales of applied deformation - representing the collective spatial mismatch (Sect. 2.6) and measure the biopsy metrics for three models: the best performing IL-initialised RL model, its corresponding IL model and an RL model. This set of experiments investigates the biopsy performance of the trained models, the IL model (without training-time deformation described above) and different RL models used for a pre-procedure planning (represented by small or no intra-procedure mismatch), and when applied to intra-procedure planning (with larger observed intra-procedure mismatch) during test, to quantify the potential value in using RL for the latter.

## Results

### Comparison between IL models and RL agents

The biopsy performance metrics are summarised in Table 1 for the two IL-models, $\pi _\textrm{Centre}$ and $\pi _\textrm{Edge}$, and three RL-trained models. IL-initialised RL models significantly outperformed their corresponding IL models (p-values $p=0.042$ and $p<0.001$ in terms of HR, for centre and edge cases, respectively), which demonstrates the added benefit of training using RL. When comparing the RL-trained methods, the best performing method is $\pi _\textrm{Centre}+RL$, with p-values $<0.020$, compared with both $\pi _\textrm{Edge}+RL$ and RL alone for HR. It also obtains the highest CCL, N.CCL and CCL coeff when compared with all other methods. This suggests that initialisation using a centre-based strategy followed by training with RL can achieve the highest performance compared to using RL and IL alone. The policy $\pi _\textrm{Edge}$ has the highest N.Coverage, which suggests a more spread strategy, but performs the worst in terms of HR and CCL. From this, a trade off is observed between HR and N.Coverage; although a larger area of the lesion could be detected, the overall effective HR is reduced.

**Fig. 2:**
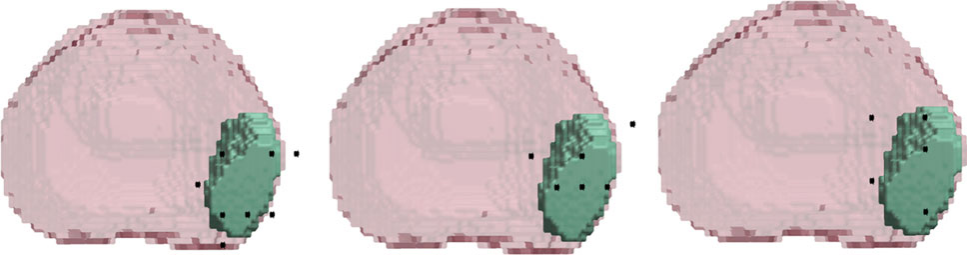
Comparison of different strategies for the same patient and lesion case. From left-right: *RL*, $\pi _\textrm{Centre}+RL$ and $\pi _\textrm{Edge}+RL$. Fired needles are illustrated in black

The difference between the three RL strategies are illustrated in Fig. 2. We observe that the initialised strategies resemble the imitated strategies shown in Fig. 1. A randomly-initialised RL strategy can be seen to cover more of the entire lesion, in contrast to $\pi _\textrm{Centre}+RL$ where only the centre section of the lesion is captured, which supports results seen in Table 1. However, a higher HR is also observed as a result of this strategy, when compared to a more spread strategy displayed by $\pi _\textrm{Edge}+RL$.

### Performance with varying intra-procedure mismatch

**Fig. 3:**
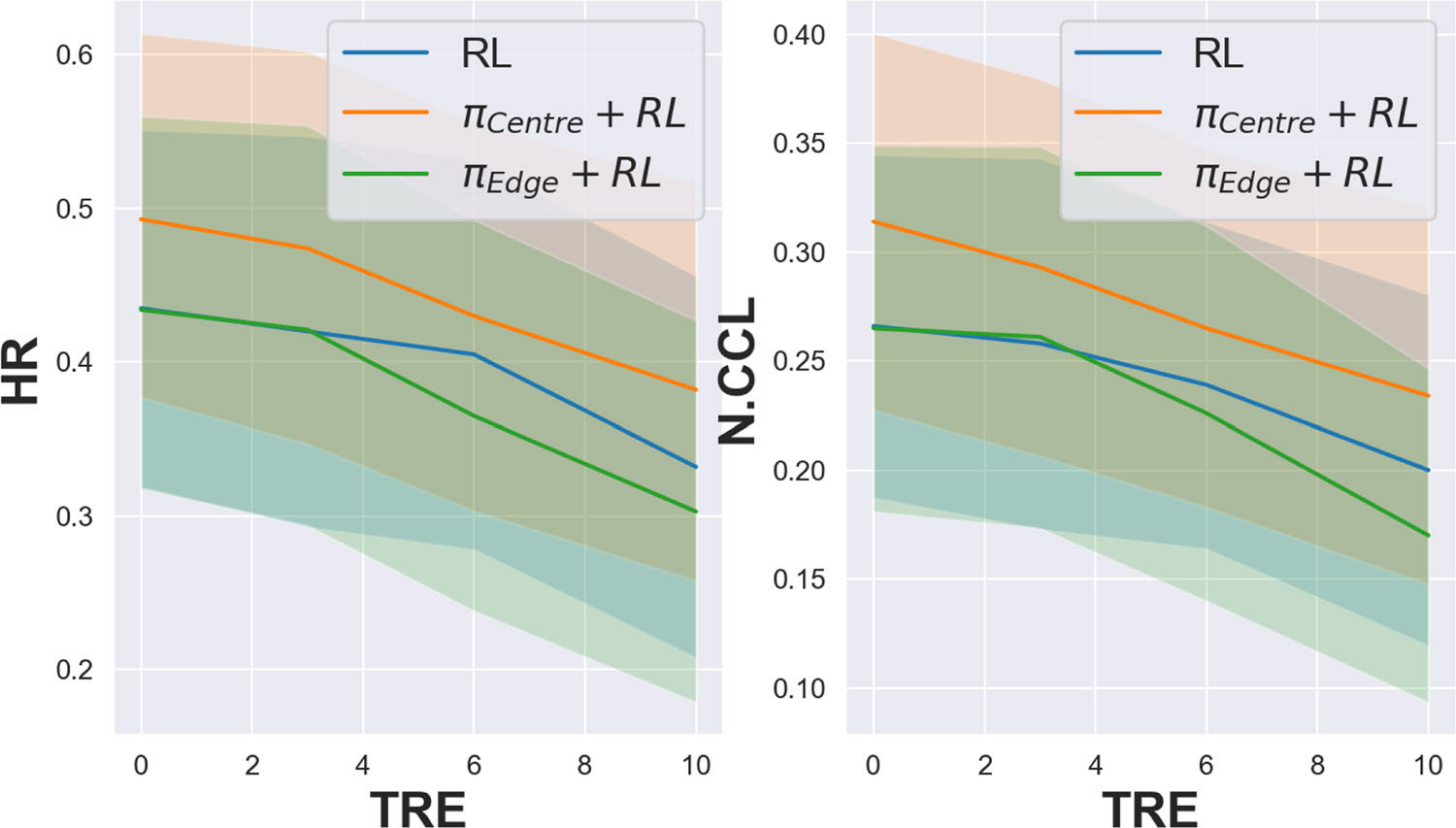
Plots of metrics (HR and N.CCL) vs TRE (in mm) for three different strategies

**Table 2:** Biopsy metrics for three RL models for varying levels of target registration error

TRE (mm)	Model	HR	N.CCL
0	RL	0.435 ± 0.232	0.266 ± 0.157
	$\pi _\textrm{Centre}$+RL	0.493 ± 0.242	0.314 ± 0.174
	$\pi _\textrm{Edge}$+RL	0.434 ± 0.253	0.265 ± 0.168
3	RL	0.420 ± 0.254	0.262 ± 0.171
	$\pi _\textrm{Centre}$+RL	0.474 ± 0.257	0.293 ± 0.173
	$\pi _\textrm{Edge}$+RL	0.421 ± 0.266	0.261 ± 0.175
6	RL	0.405 ± 0.253	0.239 ± 0.150
	$\pi _\textrm{Centre}$+RL	0.430 ± 0.251	0.265 ± 0.164
	$\pi _\textrm{Edge}$+RL	0.365 ± 0.255	0.227 ± 0.172
10	RL	0.332 ± 0.248	0.200 ± 0.161
	$\pi _\textrm{Centre}$+RL	0.382 ± 0.268	0.234 ± 0.173
	$\pi _\textrm{Edge}$RL	0.302 ± 0.251	0.169 ± 0.153

From Table 2, an expected result is obtained: the performance of the three models decreases with increasing TREs. The pattern of this decrease can be seen visually in Fig. 3. The within-model reductions due to varying TREs are statistically significant after TRE$\ge$6 mm and $\ge$10 mm, for N.CCL and HR, respectively. From [15], an average TRE of $\sim$4 mm was reported when using computational registration methods, which may suggest that RL-learned strategies are robust to intra-procedure registration errors in this application. When comparing the three strategies, we observe that $\pi _\textrm{Centre}$+RL obtains the highest HR and N.CCL, with statistical significance of $p<0.03$ for all TRE except for 6 mm. However, there is no statistical significance detected between $\pi _\textrm{Edge}$+RL and RL, which is also evident in Fig. 2, showing a similar spread strategy between the two.

**Table 3:** The HR, N.CCL values for three strategies with deformation P-values are reported between *RL* and the other two methods

		** HR**				
Scale	Rate	RL	$\pi _\textrm{centre}+RL$	$\pi _\textrm{centre}$	$p_\textrm{RL}$	$p_{IL}$
0.10	0.25	0.340 ± 0.233	0.311 ± 0.256	0.313 ± 0.258	0.165	0.198
0.50	0.50	0.335 ± 0.271	0.277 ± 0.243	0.273 ± 0.250	0.009	0.006
1.00	1.00	0.290 ± 0.272	0.233 ± 0.245	0.206 ± 0.241	0.013	<0.001

		** N.CCL**				
Scale	Rate	RL	$\pi _\textrm{centre}+RL$	$\pi _\textrm{centre}$	$p_{RL}$	$p_{IL}$
0.10	0.25	0.250 ± 0.177	0.196 ± 0.148	0.239 ± 0.170	0.502	0.458
0.50	0.50	0.196 ± 0.169	0.162 ± 0.243	0.176 ± 0.142	0.005	0.133
1.00	1.00	0.173 ± 0.165	0.128 ± 0.142	0.119 ± 0.142	0.001	<0.001

**Fig. 4:**
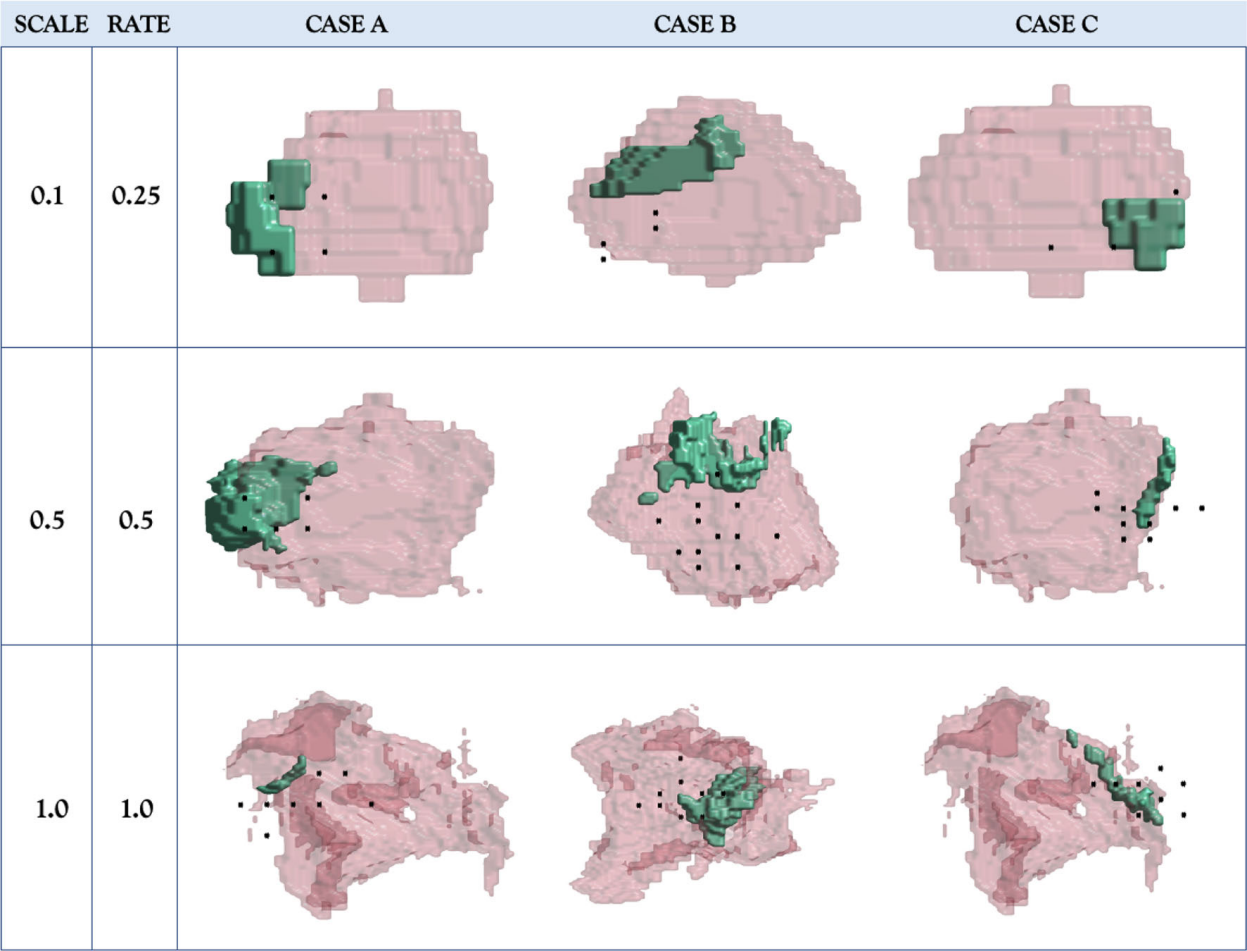
Visualisation of strategies learned by *RL* for three different cases, under varying levels of deformation (with changing scale and rate). The prostate is in pink, the lesion in green whilst black dots are needle points fired by the agent

From Table 3, an interesting phenomenon is observed: when comparing the two metrics, HR and N.CCL, a randomly-initialised agent performs better than an agent initialised with IL, which is opposite to the result in Sect. 4.1; The performance differences are statistically significant, better for larger simulated deformations, which suggests that training with RL, without using imitated actions, enables more robust learning strategies in the presence of intra-procedure mismatch. Although initilisation with IL-learned strategies in general improves the RL training, it may also adversely impact the exploration and versatility inherent in RL algorithms, especially in an increasingly complex environment. Compared with the IL models, RL yielded the same advantages as intra-procedure mismatch increased (with statistical significance found in both metrics when scale and rate>0.5). Visual results of learned strategies from the RL agent can be seen in Fig. 4. The shape of both lesion and prostate gland are visibly different when increased levels of deformation rate and scale are applied. For the last row of figures where both deformation rate and scale are 1.0, this illustrates the large range of the deformation parameters used in the experiments, with the unlikely extreme parameter values leading to arguably implausible deformation. Despite the large levels of deformation and positional changes in both prostate gland and lesion, the agent is still able to fire needles that hit the target lesion. These results highlight the strength of RL in its ability to adapt to dynamic changes, such as the frequently encountered significant deformation in prostate gland, making it a useful tool for intra-procedure planning.

## Discussion and conclusion

In this work, we describe the use of RL for both pre-procedure and intra-procedure planning. Experimental results, based on a large real clinical imaging data set, suggest that learning from demonstrations of clinically applied strategies, when combined with RL, can improve biopsy performance in terms of clinical metrics. Additionally, we find that these strategies perform well despite the presence of registration errors. While IL-initialised RL strategies prove helpful when performing pre-procedure planning, a different story is observed when the encountered intra-procedure deformation increases: strategies learned using RL alone outperforms models initialised with IL. As shown in our experiments, the actions of RL adapt based on observed changes of environment state, for instance the shape and position of the prostate and lesions, which further supports its use as an intra-operative planning tool.

When comparing to previous works in this field, we show that building on a centre-based strategy, aiming at the closest grid points near the centre of the lesion, we achieve higher HR and N.CCL for pre-procedure planning but that RL, which spreads the needles around the peripheral of the lesion, suits better for intra-procedure planning. This supports the suggestions made by [5] that strategies should also consider aiming around the edges of the lesion. Building on previous work in [6], we train models that can generalise to unseen patients, rather than training patient-specific models for each new patient. Outside of this clinical application, our work is similar to [7] where the procedure is simulated using 3D masks derived from real patient data. However, we include the modelling of intra-procedure errors that enables adaptive planning, which is not discussed in their work. Similar to [8] we include uncertainties in our model to account for errors that could occur in the real-world procedure; however, we believe that modelling individual components of these errors (such as registration and deformation) could be more informative than using a failure probability accounting for all movement errors which is done in their work.

Despite these interesting findings, our experiments are mainly limited by a number of assumptions, including the limited test of different IL models (e.g. for estimating performance change due to different number of needles feasibly required), simplified registration error based on independent errors on target and anatomy locations, and the nonrigid yet general-purpose spline-based motion models. Although these approximations are arguably necessary before any prospective studies or even interaction data acquisition (as discussed in Sect. 1.3), we aim to take the next step in acquiring real-world interaction data for developing and validating these RL-trained intra-procedure planning agents. This is much encouraged by the results presented in this study. In addition, we are also developing a virtual environment through a game-like interface for acquiring additional interaction data, by recruiting volunteers to play the biopsy game, with the under-development code available also on GitHub: BiopsyGame. Using real interaction data can enable us to compare the detection rates achieved with RL-trained models beyond simulations, which can further demonstrate the robustness of the targeted biopsy strategies.

## Footnotes

1 In general, we consider an on-policy training in this application for needle placement to be infeasible due to patient risk and other ethical concerns.
